# Ectopic Osteoid and Bone Formation by Three Calcium-Phosphate Ceramics in Rats, Rabbits and Dogs

**DOI:** 10.1371/journal.pone.0107044

**Published:** 2014-09-17

**Authors:** Liao Wang, Bi Zhang, Chongyun Bao, Pamela Habibovic, Jing Hu, Xingdong Zhang

**Affiliations:** 1 State Key Laboratory of Oral Diseases, West China Hospital of Stomatology, Sichuan University, Chengdu, China; 2 The Affiliated Hospital of Stomatology, Chongqing Medical University, Chongqing, China; 3 Department of Tissue Regeneration, MIRA-Institute for Biomedical Technology and Technical Medicine, University of Twente, Enschede, the Netherlands; 4 National Engineering Research Center for Biomaterials, Sichuan University, Chengdu, China; Faculdade de Medicina Dentária, Universidade do Porto, Portugal

## Abstract

Calcium phosphate ceramics with specific physicochemical properties have been shown to induce *de novo* bone formation upon ectopic implantation in a number of animal models. In this study we explored the influence of physicochemical properties as well as the animal species on material-induced ectopic bone formation. Three bioceramics were used for the study: phase-pure hydroxyapatite (HA) sintered at 1200°C and two biphasic calcium phosphate (BCP) ceramics, consisting of 60 wt.% HA and 40 wt.% TCP (β-Tricalcium phosphate), sintered at either 1100°C or 1200°C. 108 samples of each ceramic were intramuscularly implanted in dogs, rabbits, and rats for 6, 12, and 24 weeks respectively. Histological and histomorphometrical analyses illustrated that ectopic bone and/or osteoid tissue formation was most pronounced in BCP sintered at 1100°C and most limited in HA, independent of the animal model. Concerning the effect of animal species, ectopic bone formation reproducibly occurred in dogs, while in rabbits and rats, new tissue formation was mainly limited to osteoid. The results of this study confirmed that the incidence and the extent of material-induced bone formation are related to both the physicochemical properties of calcium phosphate ceramics and the animal model.

## Introduction

Bone defects caused by tumor resection, trauma, infection and congenital anomalies remain an important challenge in the field of orthopaedic and craniofacial surgery. Clinical treatment of such defects involves the use of natural grafts, like autograft and allograft, or synthetics [1], [2]. Autograft is still considered the gold standard for bone substitution, owning to the absence of immunogenic reaction, excellent osteogenicity, osteoinductivity and osteoconductivity. However, shortage of donor supply, persistence of pain, nerve damage, fracture, infection and cosmetic disability at the donor site remain important drawbacks. The use of allograft, where availability is less of a problem, suffers from clinical risks of disease transmission and immunogenic reaction. Another major drawback of allograft is its inconsistent quality from both a biological and a mechanical perspective [1], [3].

Tissue engineering is defined as an interdisciplinary field that applies the principles of engineering and life sciences toward the development of biological substitutes that restore, maintain, or improve tissue function or a whole organ [4]. Along with stem cell therapy that is currently being used for the treatment of various diseases and disorders [5]–[8], tissue engineering approaches have been developed and tested for various clinical applications. In orthopedics and cranio-maxillo-facial surgery, tissue engineering is considered a promising method to replace the autograft and allograft and it developed rapidly in the last two decades as a result of progress in cell biology and material science research [9]–[11]. In tissue engineering, constructs are built consisting of degradable biomaterials that serve as scaffold and transplanted (stem) cells that adhere, proliferate and differentiate on the scaffold, and, in some cases, additional growth factors to control cell fate [12], [13]. Independent of the cell sources used in bone tissue engineered constructs, one always faces the challenges of limited cell availability, and in the case of allogeneic cells, risk of infection, immunogenic reaction and disease transmission [12]. Furthermore, the complex procedure of cell harvesting, expansion and maintenance makes the tissue engineering time-, money- and labor-consuming [3]. Cell survival and vascularization are similarly challenging issues, especially when attempting to create large tissue constructs, despite developments of various *in vitro* bioreactors [14]–[17]. In 2004, Warnke et al. reported a novel method for repairing a defect in human mandible by *in vivo* tissue engineering, where the patient’s body served as a natural bioreactor. By combining ceramic particles, recombinant human Bone Morphogenetic Protein 7 (rh-BMP-7) and autologous bone marrow cells inside a titanium mesh, a sufficiently large piece of bone tissue was successfully grown inside the *latissimus dorsi* muscle of the patient prior to transplantation to repair the mandible defect [17], [18].

Calcium phosphates ceramics are extensively used as natural bone graft substitutes owning to their similarity to the mineral phase of natural bone, absence of immunogenic reactions, excellent biocompatibility and osteoconductive potential [19]. In the past five decades, a large number of publications have also shown osteoinduction by a range of calcium phosphate biomaterials, demonstrated by *de novo* bone formation upon implantation in ectopic sites, such as in the muscle of baboon [20]–[22], goat [23], sheep [19], [24]–[26], dog [21], [27]–[30], rabbit [21], [31], [32], rat [28] and mouse [33], [34] and in the subcutis of dog [28], [35], goat [36] and mouse [32]. The osteoinductive properties of calcium phosphate ceramics put forward a new idea for *in vivo* bone tissue engineering in which bone graft could be constructed *in vivo* by implanting osteoinductive calcium phosphate ceramics, without the addition of growth factors and/or stem cells in the muscle or subcutis of patient for several weeks, followed by the transplantation of the formed tissue to repair the bone defect. By doing so, the step in which cells from the patient are harvested becomes unnecessary. To demonstrate the feasibility of this method, our group has implanted an osteoinductive calcium phosphate ceramic in the femoral muscle of dogs for 8 weeks, followed by transplantation to a bone defect created unilaterally in the dog mandible to simultaneously support a dental implant. 8 weeks after transplantation, the graft fused well with the host bone, and a satisfactory osseointegration was achieved between the implant and the graft [37]. It has furthermore been shown that osteoinductive ceramics have the potential to complete the process of regeneration of large, critical-sized bone defects as successfully as autograft can [24], suggesting that even the step of intramuscular implantation prior to transplantation to bone defect may not be needed.

Osteoinduction of a great number of materials has been studied in the past five decades, using different animal species, however the exact parameters influencing and mechanisms governing ectopic bone formation are still not fully understood. It is suggested that the important parameters for successful osteoinduction are (1) chemical composition of the material and related dissolution properties, which create a microenvironment rich in calcium and phosphate ions; (2) geometry (i.e. porosity, interconnectivity and pore size), which aid nutrition and oxygen supply and recruitment of stem cells; (3) surface topography and micropores, which determines the specific surface area and affects the osteogenic differentiation of undifferentiated cells either directly (adhesion mediated differentiation) or indirectly (via adsorption/precipitation of relevant endogenous proteins); (4) the species and genetic background of animal models; and (5) implantation site [21], [23], [24], [38]–[42].

Previous studies have demonstrated that osteoinduction by biomaterials predominantly occurs in large animals [19]–[36], [39], [43], while in small animals the incidence of ectopic bone formation is relatively low [32]–[34]. The reason for these differences between animal species is still unclear. Experiments in large animal models are associated with issues of limited availability, high cost and ethical concerns, which is why studies in large animals usually involve relatively small sample size, limiting their reliability. Testing osteoinductivity of biomaterials in small animals, such as mice and rats, would not only allow studies with a large sample size, but it would also enable more in-depth immunohistochemical analyses which are readily available for studies in small animals. Such in-depth analyses could provide more insight in biological mechanisms governing bone induction by synthetic biomaterials.

The aim of this study was to determine material parameters that are essential for osteoinduction to occur. In addition, this study is aimed at investigating influence of species on material-induced ectopic bone formation, as an attempt to find most suitable model for this type of studies.

## Materials and Methods

### Preparation and characterization of the ceramics

Raw materials as well as final calcium phosphate ceramic scaffolds were provided by the National Engineering Research Center for Biomaterials (Chengdu, Sichuan, China). Briefly, the ceramics were prepared in house using wet precipitation and H_2_O_2_ foaming method, respectively, as previously described [37]. Hydroxyapatite (HA) was sintered at 1200°C (HA1200) and biphasic calcium phosphate (BCP), consisting of 60 wt.% HA and 40 wt.% β-tricalcium phosphate (TCP) was sintered at either 1100°C (BCP1100) or 1200°C (BCP1200) for 3 hours. Ceramic cylinders with dimensions φ5 mm×8 mm, φ4 mm×6 mm and φ2 mm×3 mm intended for implantation in dogs, rabbits and rats, respectively were cut and cleaned in ultrasonic baths. The size of implants varied per animal model as to adapt to the size of the implantation site. All the samples were sterilized by autoclaving before implantation. The chemical composition of the ceramics was analyzed by X-ray diffraction (XRD, Miniflex, Rigaku, Japan). The ultrastructure and the surface microstructure of the ceramics were investigated using scanning electron microscopy (XL30 ESEM FEG, Philips, The Netherlands). For quantification of the average macropore size and macroporosity, ceramic blocks (n = 8) were embedded in methylmethacrylate (MMA), and allowed to polymerize, after which 10–20 µm thick sections were cut using a diamond saw (Leica, Germany). Semi-automated measurements were performed using a light microscope (Nikon, Japan, Objective×10), connected to a KS400 imaging system (Carl Zeiss, Germany).

### Animal experiments

The experimental protocol was approved by the Animal Care and Use Committee of Sichuan University. To determine the minimum sample size needed to obtain statistical differences between animal species and three types of materials, the sample sizes were estimated by applying the equation for comparative studies [44]. Surgery was performed on 27 mongrel dogs (10–15 kg, male), 36 New Zealand white rabbits (2.5–3.0 kg, male) and 54 S.D. rats (200–250 g, male) at the Experimental Animal Centre of Sichuan University. After anesthesia of the animals with 3% sodium pentobarbital (Sigma Chemical, St. Louis, MO, USA), the operation sites were shaved and the skin was sterilized with iodine. A longitudinal incision (10 cm, 5 cm and 2 cm in dogs, rabbits and rats, respectively) was created and the muscles were exposed by blunt separation. The muscle incisions were made by scalpel and the separated intramuscular pockets of appropriate size, with a distance of approximately 1 cm from one another were created by blunt separation. Each pocket received a single ceramic implant, after which the wound was closed in layers by using silk suture. After surgery, penicillin was administered intramuscularly for 3 consecutive days to prevent infection. In total 108 samples of each ceramic was implanted in each of the three species: four cylinders (φ5 mm×8 mm) of each ceramic type were implanted in the dorsal muscle of each dog, three cylinders (φ4 mm×6 mm) were implanted in the dorsal muscle of each rabbit, and two cylinders (φ2 mm×3 mm) of each ceramic were implanted in the femoral muscle of each rat. Animals were sacrificed by an overdose of sodium pentobarbital 6, 12 or 24 weeks post-implantation in a randomized manner. These survival times were selected based on earlier studies on ectopic bone formation by synthetic biomaterials [34].

### Retrieval of implants, histology, and histomorphometry

At explantation, the samples were collected with surrounding tissues and fixed in 4% buffered formaldehyde solution. The fixed samples were then decalcified in 10% ethylene diamine tetraacetic acid (EDTA) for 15 days and washed with phosphate buffer solution (PBS), dehydrated with gradient of ethanol solutions at 70%, 80%, 90%, 95% and 100%×2, and then embedded in paraffin. Continuous 5 µm sections were made parallel to the long axis of the implants and then stained with hematoxylin and eosin (HE) (for all time points) or Masson’s trichrome staining (for 12 weeks, which is representative for other time points) for histological analysis. Occurrence of osteoid tissue formation was assessed by three experienced pathologists separately without knowledge of the source of the specimens.

Histomorphometry was performed on 25 randomly selected fields at a magnification 10× (objective) of a single section taken from the middle of each implant, by using a light microscope (Nikon, Japan) coupled to a KS400 imaging system (Carl Zeiss, Germany). The macropore area and the area of bone or osteoid tissue were semi-automatically measured and the percentage of the induced bone and osteoid tissue inside the macropores (b% and o% respectively) were calculated. While b% was determined in all samples in which ectopic bone formation was induced, o% was determined in samples where osteoid, but no bone formation was found. The reason for this is the fact that, while pathologist can easily distinct between osteoid and bone, it is very difficult to draw the line between the area where the osteoid stops and bone tissue starts, making a reliable histomorphometry impossible to perform. Therefore, in samples with both osteoid and bone, the total area was used to calculate %b, whereas only the samples without bone were used for calculating %o. This makes results on bone and osteoid incidence and %b and %o complementary.

### Statistical analysis

Light micrograph images were analyzed with IPP (Image-Pro-Plus) software, and statistical analysis was performed on histomorphometry data using SPSS19.0. Pearson’s chi-square test was conducted to compare the overall constituent ratios of bone and osteoid tissue incidence in the three groups and differences with p<0.05 were considered significant. The adjusted test of level (α’) was used to compare the constituent ratios of each two groups and the differences with p<0.017 were considered significant (Partitions of chi-square method) with α’ = 2α/k(k–1) k = 3, α = 0.05.

## Results

### Properties of the ceramics

**Figure 1:**
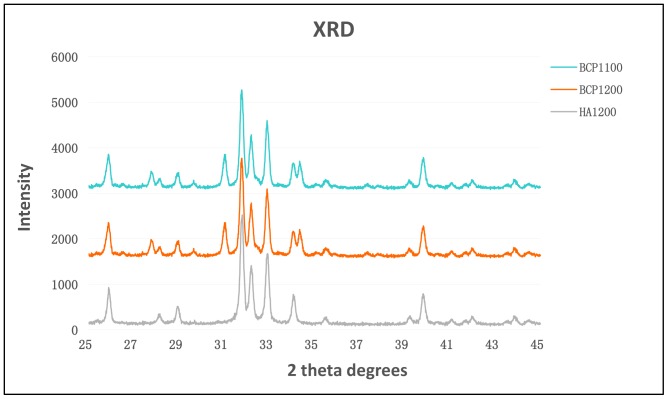
XRD patterns of the three ceramics. The patterns of BCP1100 and BCP1200 demonstrated biphasic nature of the ceramics consisting of HA and β-TCP. No apparent differences were found as a result of difference in sintering temperature. The pattern of HA1200 was typical of phase-pure hydroxyapatite.

**Figure 2:**
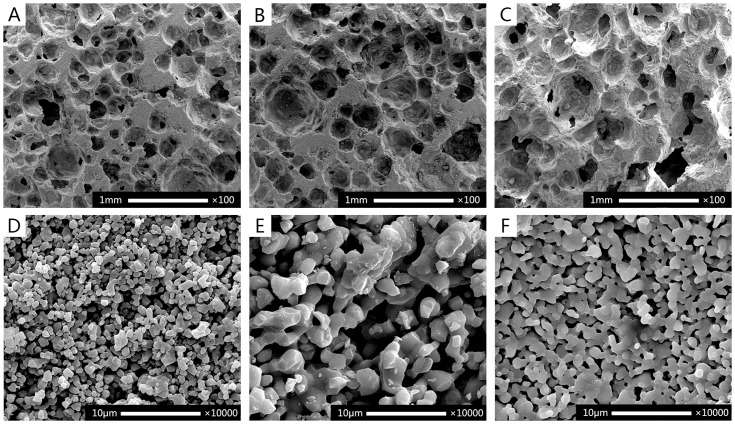
SEM micrographs showing macrostructure (A–C) and surface microstructure (D–F) of BCP1100 (A,D), BCP1200 (B,E) and HA1200 (C,F). All ceramics were porous with similar macroporous structure (A–C). The surface of the BCP1100 (D) ceramic exhibited smaller grains and a larger number of micropores than the chemically identical BCP1200, that was sintered at 1200°C (E). Grain size and number of micropores in HA1200 were between those of BCP110 and BCP1200 (F), which is in accordance with specific weight measurements in Table 1. Scale bar = 1 mm for A–C and 10 µm for D–F.

The X-ray diffraction analysis (**Fig. 1**) of BCP1100 and BCP1200 demonstrated biphasic nature of the ceramics and no apparent differences were found as a result of difference in sintering temperature. The pattern of HA1200 was typical of phase-pure HA. The SEM analysis (**Fig. 2**) showed that all ceramics were porous. The pore size of macropores in BCP1100, BCP1200 and HA1200 were 380±50 µm, 400±50 µm and 400±50 µm respectively. However at the temperature of 1100°C the surface of the BCP ceramics was rougher with more micropores between the ceramic grains as compared to the ceramic sintered at 1200°C (**Fig. 2**). Characterization of the three ceramics is summarized in Table 1.

**Table 1:** Physico-chemical characterization of biphasic calcium phosphate sintered at 1100°C (BCP1100), of biphasic calcium phosphate sintered at 1200°C (BCP1200) and of phase-pure hydroxyapatite sintered at 1200°C (HA1200).

	*BCP1100*	*BCP1200*	*HA1200*
Weight ratio HA/β-TCP	60/40	60/40	100/0
Macropore size (µm)	380±50	400±50	400±50
Overall porosity (%)	70.8	65.2	66.9
Specific weight (g/cm^3^)	0.970	1.21	1.10

### Histological and histomorphometrical analyses

**Figure 3:**
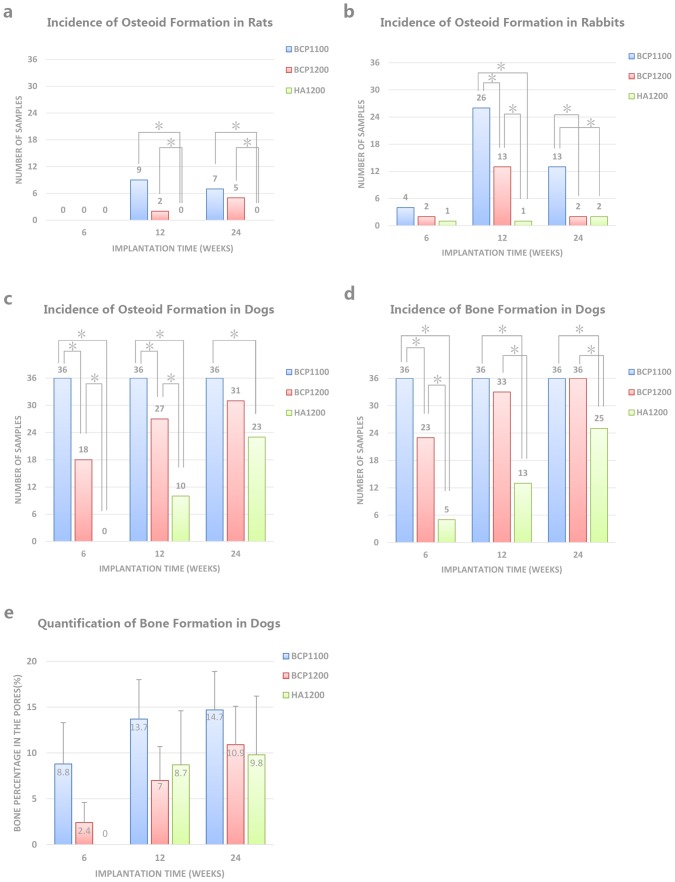
Incidence of bone and osteoid tissue formation in different animals (A–D) and percentage of bone area in pores of dogs (E) at 6, 12 and 24 after implantation. Osteoid formation in rats was not observed at 6 weeks in any of the ceramics, while at 12 and 24 weeks, only BCP ceramics showed signs of osteoid formation (A). In rabbits, all ceramics showed ectopic osteoid formation at all time points, with a highest incidence at 12 weeks. At all time points, BCP1100 showed a higher osteoid incidence than BCP1200, which was again higher than osteoid incidence in HA1200 (B). In dogs, osteoid formation was observed in all ceramics at all time points, except in HA1200 at 6 weeks. Like in rabbits, a decreasing osteoid incidence was observed from BCP11 to BCP1200 to HA1200 (C). All ceramic showed ectopic bone formation in dogs at all time points (D) following a trend that was similar to that of osteoid incidence (C). Quantification of % of ectopic bone formation in available pore area showed that, in general, BCP1100 showed higher values than BCP1200 and HA1200; however, these differences were not statistically significant (E).

There were no surgical complications and all samples were retrieved. At retrieval, all implants were surrounded by well-vascularized muscle tissue. No signs of an inflammatory tissue response directly related to the implants were observed. **Table 2** and **figure 3** provide an overview of the osteoid and bone incidence, as well as percentage of bone and osteoid tissue in the pores after 6, 12 and 24 weeks of implantation in the three animal species.

**Table 2:** A summary of tissue response to the three types of ceramics in different animals.

Species		Rats			Rabbits			Dogs		
Intervals (weeks)		6	12	24	6	12	24	6	12	24
	**Incidence of bone**	0/36	0/36	0/36	0/36	3/36	0/36	36/36	36/36	36/36
	**Percentage of bone in available pore area (b%)**	0	0	0	0	1.2±0.8	0	8.8±4.5	13.7±4.3	14.7±4.2
**BCP1100**	**Incidence of osteoid**	0/36	9/36	7/36	4/36	26/36	13/36	36/36	36/36	36/36
	**Percentage of osteoid tissue in available pore area (o%)**	0	0.96±0.31	0.40±0.23	0.40±0.27	1.22±0.26	1.57±0.51	N/A	N/A	N/A
	**Incidence of bone**	0/36	0/36	0/36	0/36	0/36	0/36	18/36	27/36	31/36
	**Bone area in available pore area (b%)**	0	0	0	0	0	0	2.4±2.2	7.0±3.7	10.9±4.2
**BCP1200**	**Incidence of osteoid**	0/36	2/36	5/36	2/36	13/36	2/36	23/36	33/36	36/36
	**Percentage of osteoid tissue in available pore area (o%)**	0	0.83±0.16	0.39±0.17	2.21±0.20	1.02±0.46	1.62±0.34	0.56±0.24	1.15±0.19	1.42±0.26
	**Incidence of bone**	0/36	0/36	0/36	0/36	0/36	0/36	0/36	10/36	13/36
	**Bone area in available pore area (b%)**	0	0	0	0	0	0	0	8.5±5.9	8.8±6.4
**HA1200**	**Incidence of osteoid**	0/36	0/36	0/36	1/36	1/36	2/36	5/36	13/36	25/36
	**Percentage of osteoid tissue in available pore area (o%)**	0	0	0	0.65±0.08	1.34±0.11	0.70±0.18	1.43±0.38	1.22±0.41	1.51±0.46

**Figure 4:**
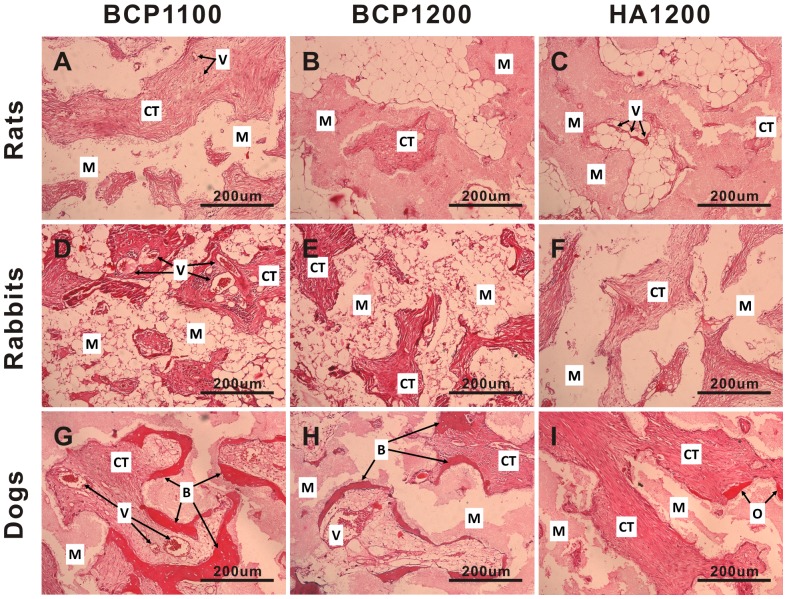
Histological evaluation of tissue formation in the three ceramics upon intramuscular implantation in rats, rabbits and dogs. Representative images showing tissue response to BCP1100 (A, D, G), BCP1200 (B, E, H) and HA1200 (C, F, I) upon implantation in the femoral muscle of rat, dorsal muscle of rabbit and dog for 6 weeks. Decalcified sections, HE Staining, magnification = ×100, scale bar = 200 µm; V→ Blood vessel, CT→Connective tissue, M→Material, O→Osteoid tissue, B→Bone.

**Figure 5:**
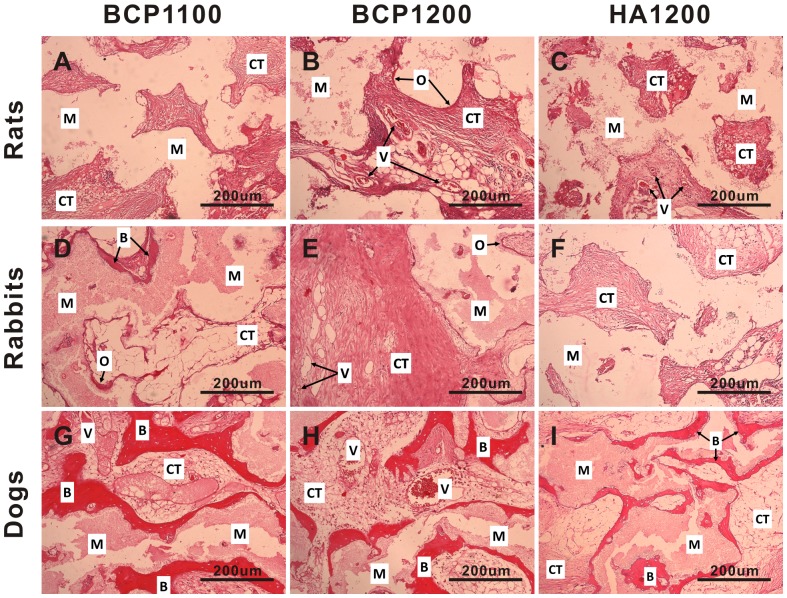
Histological evaluation of tissue formation in the three ceramics upon intramuscular implantation in rats, rabbits and dogs. Representative images showing tissue response to BCP1100 (A, D, G), BCP1200 (B, E, H) and HA1200 (C, F, I) upon implantation in the femoral muscle of rat, dorsal muscle of rabbit and dorsal muscle of dog for 12 weeks. Decalcified sections, HE Staining, magnification = ×100, scale bar = 200 µm; V→Blood vessel, CT→Connective tissue, M→Material, O→Osteoid tissue, B→Bone.

**Figure 6:**
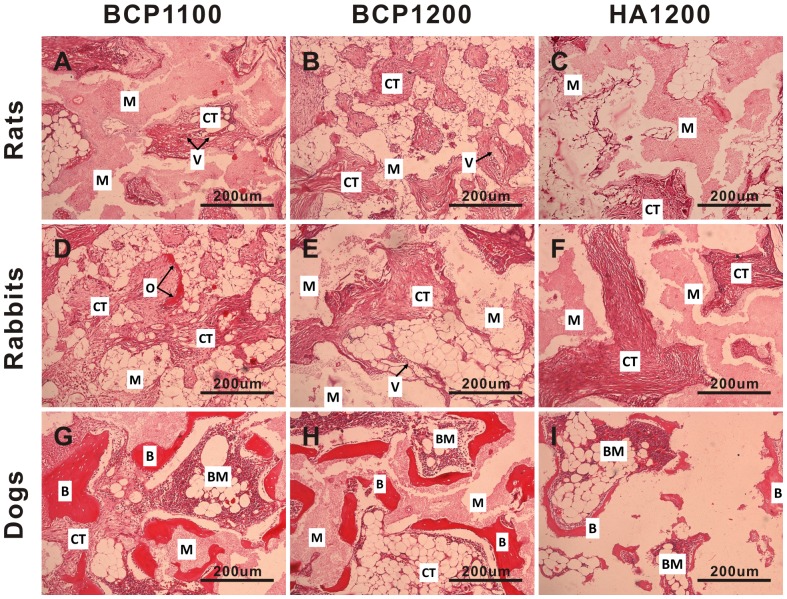
Histological evaluation of tissue formation in the three ceramics upon intramuscular implantation in rats, rabbits and dogs. Representative images, showing tissue response to BCP1100 (A, D, G), BCP1200 (B, E, H) and HA1200 (C, F, I) upon implantation in the femoral muscle of rat, dorsal muscle of rabbit and dorsal muscle of dog for 24 weeks. Decalcified sections, HE Staining, magnification = ×100, scale bar = 200 µm; V→Blood vessel, CT→Connective tissue, M→Material, O→Osteoid tissue, B→Bone, BM→Bone marrow.

In rats, no bone formation was observed in any of the three ceramics, independent of the implantation time. At 6 weeks, no osteoid was observed in any of the three ceramics (**Fig. 4A–C**), and similarly, HA1200 did not induce osteoid formation at the later time points either (**Fig. 5C** **or** **Fig. 6C**). In HA1200, the tissue in the pores was fibrous in nature, only containing fibroblast-like cells (**Fig. 4C** **,** **5C** **and** **6C**). After 12 weeks of implantation, a thin band of osteoid with some compacted cuboidal cells adjacent to pore walls was observed in 9 out of 36 samples of BCP1100 and in 2 out of 36 samples of BCP1200. The percentage of osteoid tissue formation was relatively low (0.96±0.31% for BCP1100 and 0.83±0.16% for BCP1200 respectively). Only the difference between HA1200 and the other two ceramics was statistically significant (p<0.017). At 24 weeks, incidence of osteoid formation was 7/36 (0.40±0.23%) and 5/36 (0.39±0.17%) in BCP1100 and BCP1200, respectively, a difference that was not statistically significant, while there was a statistical difference between BCP1100 and HA1200 (**Fig. 3A** and **Table 2**).

Intramuscular implantation of the three ceramics in rabbits did not lead to a pronounced ectopic bone formation. Limited amount of bony tissue and bone trabeculae (1.2±0.8%) was only observed in three BCP1100 samples from the 12-week group, all coming from one animal (**Fig. 5D** and **Table 2**). Osteoid formation was observed in the three ceramics at all time points, although the incidence was strongly dependent on the material. In HA1200, only few samples showed osteoid formation (1/36, 1/36 and 2/36 after 6, 12 and 24 weeks respectively), and the pores of the ceramic were predominantly occupied by fibrous tissue containing fibroblasts, neutrophils, lymphocytes and plasma cells (**Fig. 4F** **,** **5F** and **6F**). Osteoid incidence was 4/36, 26/36 and 13/36 for BCP1100 and 2/36, 13/36 and 2/36 for BCP1200 after 6, 12 and 24 weeks respectively (**Fig. 3B** **,** **4D–E** **,** **5D–E** **,** **6D–E** and **Table 2**). In terms of osteoid incidence, no statistically significant differences were observed among the three ceramics after 6 weeks of implantation. BCP1100 showed a higher osteoid incidence than BCP1200 and HA1200 at both 12 and 24 weeks (p<0.017).

Similar to bone incidence, in rabbits, the area percentage of ceramic pores occupied by osteoid tissue was highest in BCP1100, increasing from 0.40±0.27% at week 6 to 1.57±0.51% at 24 weeks, respectively. In BCP1200 and HA1200, the %o remained below 2% at all time points, and considering the very low osteoid incidence, a statistical comparison of histomorphometrical data was not sensible (**Table 2**).

**Figure 7:**
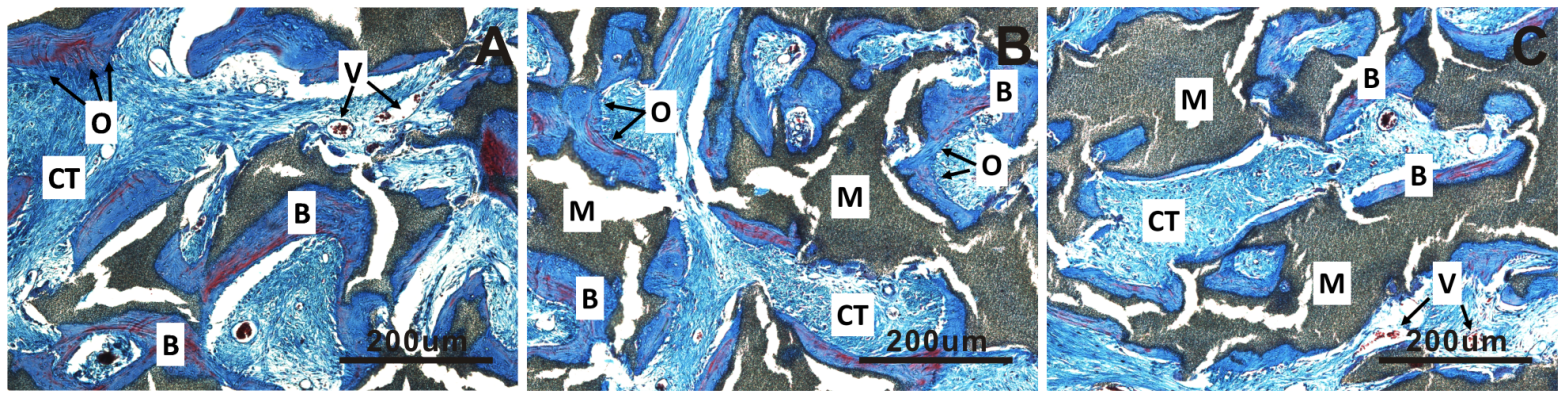
Histological evaluation of bone formation upon implantation of the three ceramics in dorsal muscles of dogs for 12 weeks. The highest amount of osteoid and ectopic bone were observed in BCP1100 (A), followed by BCP1200 (B) an HA1200 (C). In all cases, bone was trabecular in appearance, with laminar bone and osteocytes contained in the lacunae, and infiltrated by blood vessels. Decalcified sections, Masson’s trichrome staining, magnification = ×100, scale bar = 200 µm; V→Blood vessel, CT→Connective tissue, M→Material, O→Osteoid tissue, B→Bone.

In dogs, the incidence and the amount of osteoid and ectopic bone were higher for all the ceramics as compared to tissue formation found in rats and rabbits (**Fig. 3C–D** **,** **4G–I** **,** **5G–I** **,** **6G–I** and **Table 2**). In the pores of all BCP1100 samples, formation of *de novo* bone adjacent to the material surface was observed as early as at 6 weeks, and bone incidence 36/36 remained at the later time points. With time, the bone appeared more trabecular, with lacunae containing osteocytes (**Fig. 4G** **–** **6G** **and** **Fig. 7A**). In BCP1200, pores were mainly filled with star-shaped cells or thin, elongated cells at 6 weeks and bone trabeculae were observed in a half of the samples (**Fig. 4H**). After 12 weeks, bone trabeculae were observed in the pores of the ceramic in most samples (27/36), with a layer of osteoblasts aligning bone (**Fig. 5H** **and** **Fig. 7B**). At week 24, bone incidence further increased to 31/36 and maturation of the trabeculae and the formation of new blood vessels, along with appearance of myeloid tissue were observed (**Fig. 6H**). The osteoinductive potential of HA1200 was the poorest among the three ceramics, without bone formation at week 6, and with pores filled with fibrous tissue mainly consisting of fibroblasts or fibroblast-like cells. Bone formation was observed after 12 weeks in 10/36 samples (**Fig. 5I** **and Fig, 7C**) and incidence further increased to 23/36 after 24 weeks (**Fig. 6I**).

Incidence of ectopic bone formation in BCP1100 was statistically higher than in HA1200 at all time points. Further, statistical differences in bone incidence were observed between BCP1200 and HA1200, and also between BCP1100 and BCP1200 after 6 and 12 weeks. Regarding presence of osteoid tissue, HA1200 performed significantly poorer than the other two ceramics at all time points, while no significant differences between BCP1100 and BCP1200 were observed after 12 and 24 weeks of implantation.

In BCP1100, the percentage of bone in the pores increased from 8.8±4.5% at week 6 to 13.7±4.3% at week 12 and 14.7±4.2% after 24 weeks (**Fig. 3E** **and** **Table 2**). The percentage of bone in the pores of BCP1200 was lower than that in BCP1100, increasing from 2.4±2.2% at week 6 to 7.0±3.7% and 10.9±4.2% at 12 and 24 weeks, respectively. Finally, the bone tissue area in the pores of HA1200 was 8.5±5.9% and 8.8±6.4% after 12 and 24 weeks, respectively. The area of osteoid tissue inside the pores of samples that did not contain bone (for BCP1100 and BCP1200) was always below 1.5%.

## Discussion

In the present study, we explored the dependence of osteinductive potential of three calcium phosphate bioceramics on their physicochemical properties and on the animal model in which osteoinduction was studied. The three ceramics varied in their chemical composition, microstructure or both as these properties have previously been suggested to be of influence on the osteoinductive potential [3], [26], [32], [39], [41], [45]. To investigate the effect of chemical composition, both phase-pure HA and biphasic calcium phosphate ceramic consisting of HA and TCP were used. In addition, BCP ceramic was sintered at either 1100°C or 1200°C, to vary surface microstructure in terms of grain size, microporosity and surface area. The three ceramics were implanted intramuscularly in rats, rabbits and dogs to obtain information about the species-dependent ectopic bone formation. While a number of studies exists in which synthetic biomaterials were tested for their osteoinductive potential in various animal models, in most of these studies one animal species was used as a model, making the comparison among different studies difficult. In studies where different animal species were used in parallel, either a low number of animals per time point was used [46] or bone formation was assessed at a single time point [32]. Besides variation among different species, variations in the amount of bone induced in individuals from the same species have been reported, making the sample size of such studies of great importance [23]. Earlier studies have shown that experiments involving 8–12 large animals (goats, sheep, dogs) allow for a reliable comparison of ectopic tissue formation by different materials [24]. Based on these studies, and using a formula to estimate the minimum sample size for our study design [44], we have selected the sample size for the present study.

Physicochemical properties of osteoinductive materials such as chemical composition, geometry, surface microstructure and implant size have been reported as critical parameters for successful osteoinductivity. As recently reviewed [39], in the past decades, a large number of publications have illustrated osteoinduction by diverse biomaterials, such as synthetic and coral-derived HA ceramics, α-TCP, β-TCP, BCP and calcium phosphate cements (CPC). Besides calcium phosphate-containing biomaterials, osteoinduction was also observed in alumina ceramic, titanium and glass ceramics. However, so far most biomaterials shown to be osteoinductive contained calcium phosphate, which suggests the importance of calcium and phosphate ions in the process of osteoinduction by biomaterials [47]–[51]. When a biphasic calcium phosphate ceramic, consisting of HA and β-TCP, was compared with a pure HA ceramic with similar macro- and microstructure in a goat model, more bone was found in BCP ceramic. This suggested that the more soluble calcium phosphate phases are more osteoinductive than the less soluble ones [3]. These results are in accordance with our findings, where the biphasic ceramics (BCP1100, BCP1200) showed significantly more osteoid tissue and/or bone than the phase-pure HA (HA1200) in dogs (**Fig. 3** **–** **7** and **Table 2**). Furthermore, initiation of ectopic bone formation occurred earlier in biphasic than in HA ceramic in dogs. While in rats and rabbits no bone formation was found in any of the ceramics, the incidence of osteoid tissue formation, believed to precede bone formation was also more prominent in biphasic ceramic than in HA ceramic. The proposed reasons for the observed positive effect of the more soluble calcium phosphate ceramic on ectopic bone formation are multiple, ranging from the sole effect of calcium release of osteogenic differentiation of cells, to the process of dissolution/reprecipitation of the biological apatite layer on the ceramic surface, accompanied by the co-precipitation of endogenous osteoinductive growth factors. In addition, the inflammatory response (especially the chronic inflammation) induced by the implanted ceramics and its solubility products (i.e. cytokines) may also be an initiator of material-induced ectopic bone formation [39], [52]–[55].

Besides chemistry, structural features of the ceramics, at macro- and micro level were suggested to influence osteoinductive potential of biomaterials. For example, Ripamonti showed that geometry is one of the crucial characteristics necessary for rendering a biomaterial osteoinductive [20] and presence of interconnected macropores was suggested to positively influence ectopic bone formation by biomaterials [24], [56], [57]. In addition to macroporosity, surface features like size of grains and presence of micropores have also been proven important in the bone induction process [58]–[61]. Micropores enlarge the surface area, which facilitates ion exchange and bone-like apatite formation, and/or accompanied binding of endogenous bone inducing proteins to the surface, all of which may positively affect the adhesion, proliferation, and differentiation of the bone forming cells [23], [62]. Furthermore, the microstructure of the materials has been shown to directly influence cell shape and related signaling pathway, and therewith also the final cell fate [63]–[65]. In the present study, BCP sintered at a relatively low temperature of 1100°C (BCP1100), having a larger specific surface area as well as more micropores than BCP and HA sintered at 1200°C (BCP1200 and HA1200), indeed showed relatively more bone formation than the other two ceramics.

As previously mentioned, osteoinduction by biomaterials has shown large interspecies differences, and numerous studies have demonstrated that material induced bone formation is more likely to occur in large animals [19]–[36], [39], [43] such as dogs, goats, sheep, and non-human primates, than in rabbits [31], [32], rats and mice [32]–[34], as shown by Kurashina et al. [31] and Yuan et al. [32] and confirmed by our study. While no bone formation was observed in rats by any of the implanted materials, and bone formation in rabbits was limited to BCP1100 and only a single animal, osteoinduction in dogs occurred as early as after 6 weeks of implantation in all the ceramics tested, though to a different extent as demonstrated by histomorphometry (**Table 2**). Osteoid formation occurred more frequently than bone formation, however, its incidence was also largely dependent on both the ceramic type and the species, following a trend similar to that of bone formation dependence on material and species parameters: increasing from rat to rabbit to dog for all ceramics and from HA1200 to BCP1200 to BCP1100 in all animals. While incidence of osteoid was in general higher than incidence of bone formation, percentage of osteoid tissue inside the pores of the ceramics was rather limited, though it should be mentioned that this analysis was only performed on those samples that did not show any bone formation. Based on these findings, it may be speculated that osteoid formation is a precursor of bone formation during osteoinduction by biomaterials, which in turn suggests surface dynamics of dissolution and reprecipitation to play an important role in this process.

The difference in the species-dependent osteoinductive potential of the materials is not clear as yet, but it could plausibly be related to various factors, such as genetic background and related physiological metabolism of each species, including different substance exchange, degradation and absorption kinetics, levels of BMPs or other osteogenic factors available near the implants and differences in response to osteogenic signals. In a recent study by Barradas et al. [34], in which 11 mouse strains were compared, reproducible ectopic bone formation upon subcutaneous implantation of calcium phosphate ceramics was only found in one strain (FVB mice), indeed suggesting the influence of genetic factors. Besides interspecies variations in osteoinductive potential, also differences in ectopic bone formation among individual animals of the same species have been found in this study, comparable to the differences among individuals reported earlier [23]. Both the differences in the response of the species and the response of the individuals from the same species may have important implications for the clinical use of these synthetic bone graft substitutes as alternatives to natural bone grafts. The growing understanding of the parameters that influence osteoinductive potential of biomaterials can be used to in design new materials, which may be osteoinductive in smaller animals, like rats and mice as well.

With this study, in which we systematically analyzed effects of physicochemical properties of calcium phosphate bioceramics in three animal species and by using large numbers of samples, we have shown that both presence of a more soluble β-TCP phase in the ceramic and a decrease of sintering temperature that affects surface microstructure of the ceramic positively influence its ability to induce ectopic osteoid and bone formation. Furthermore, we have demonstrated that material-induced ectopic bone formation indeed reproducibly occurred in dogs, while in rats and rabbits, only osteoid formation was observed.

## Conclusion

The results of the present study showed that a BCP ceramic, consisting of HA and TCP, that was sintered at a temperature of 1100°C showed more osteoid and/or bone formation upon ectopic implantation than BCP (or HA) sintered at a higher temperature (1200°C). Concerning the animal species, all tested materials showed more ectopic osteoid and bone formation in dogs than in rabbits or rats. In conclusion, this study demonstrated that both, the type of calcium-phosphate ceramic and the animal species influence the osteoinductive potential of the bioceramics.
